# Using Basic Ethical Principles to Evaluate Safety Efforts in Transfusion Medicine

**DOI:** 10.1155/2012/407326

**Published:** 2011-12-07

**Authors:** Jay P. Brooks

**Affiliations:** Department of Pathology, Immunology and Laboratory Medicine, College of Medicine, University of Florida, P.O. Box 100275, Gainesville, FL 32610-0275, USA

## Abstract

Pursuit of pharmaceutical purity of the blood in the bag has led to a shrinking donor base and a significantly more expensive product. Decisions regarding new infectious marker testing and donor deferrals have typically been made emphasizing decreasing one specific risk without considering the effect the intervention will have on the overall safety and availability of blood transfusion. Regulations have been formulated by governmental agencies with limited input from the medical community. The decision making process has lacked risk benefit analyses and has not had the robustness associated with spirited discussions. Policies made in this manner may result in certain risks being decreased but can also have adverse unintended consequences. Being guided by the ethical principles of nonmaleficence, beneficence, autonomy, and justice, we need to evaluate our actions in the context of overall blood safety rather than narrowly focusing on any one area.

## 1. Introduction

If automakers concentrated on perfecting the braking system in new cars while ignoring possible improvements in passenger restraint systems and steering mechanisms, consumers would likely question their wisdom and demand that they redirect their efforts to improve overall automotive safety rather than to focus narrowly on any one component of the vehicle. While most would agree that it would be great to have better, safer brakes, if the pursuit of the ideal stopping system was at the expense of improvements in safety of other parts of the vehicle, particularly if the improvements were less costly and could result in greater overall safety benefits, consumers would balk.

In blood banking and transfusion medicine, we have been doing something akin to our hypothetical automobile manufacturers. We have concentrated on the pharmaceutical purity of the blood in the bag resulting in the diminished possibility of implementing other advances in blood transfusion safety. Evaluating the whole transfusion process from blood collection to care of the recipient during and immediately after the transfusion can provide significantly greater payoffs than the implementation of newer, better, but costlier measures to push the already low risk of viral transmission ever closer to the elusive rate of zero. There are other practices that may inadvertently decrease overall safety because of our obsession with the pharmaceutical purity of blood, and these will also be addressed.

## 2. The Zero-Sum Game

A zero-sum game is one where a player can gain only at the expense of another [1]. Cutting a cake is an example. If one takes a piece of cake, there is less cake available for others. Whether health care is a zero-sum game or not is debatable. If gains can be made in terms of productivity and not at the expense of the other players, it would be a non-zero-sum game. In some circumstances this certainly occurs. Overall, however, and certainly in the short term, I believe that health care is largely a zero-sum game. Given fixed resources, increasing expenditures for diagnosing and treating one type of illness will result in a corresponding decrease in resources available for all others. While most would favor increased funding for cancer treatment, if it resulted in decreased treatment for heart diseases, this would likely curb their enthusiasm. I maintain that funding for blood banking and transfusion medicine is largely a zero-sum game. In the US, most health care spending emanates from government agencies, mostly in the form of Medicare and Medicaid, and private insurers. Over a given period of time, reimbursements are fixed. When blood prices increase, there will not be a corresponding increase in reimbursement, at least not in the short run [2]. The price increases may be offset by reducing funding to the transfusion medicine services by decreasing staffing or by blocking implementation of new technology available to increase safety such as electronic methods of patient and blood identification or improved methods of utilization review. Hospital administrators faced with a blood bill well beyond the amounts budgeted and reimbursed will likely be reluctant to fund new activities in the transfusion medicine service.

## 3. How We Have Allocated Resources

The ethical principle of justice is concerned with how we should allocate scarce resources. After years of relatively modest increases in the prices of blood, hospitals saw their costs nearly double between 1999 and 2004, with the increases being related primarily to the addition of blood safety measures [3]. In the US, blood services and the FDA were viewed by many as having been slow to act when it became evident that HIV was being transmitted by blood transfusion [4]. The outrage showered upon these entities by the media and the public sensitized them to be diligent, arguably overly diligent, in shielding the blood supply from infectious diseases. HIV is unique among transfusion hazards; AIDS has received more press coverage and has been the subject of more books, movies, and television dramas than any other infectious illness in recent history. The public has developed an understandable dread of acquiring HIV through blood transfusion. The course of the disease has been portrayed as horrific: a death sentence preceded by unusual suffering. When HIV testing of donated blood was introduced in 1985, the prevalence of HIV in some communities was as high as 1 in 100 [5]. The HIV antibody test was sensitive and extremely effective, dramatically reducing the number of cases of transfusion-transmitted HIV. For the five years following the introduction of the HIV antibody test, there were only five cases of transfusion-associated HIV per year reported to the Centers for Disease Prevention and Control [6]. At a cost of $3,600 per quality-adjusted life year gained (QALY), the test was a true bargain [7]. A typical cutoff for QALY in health care is $50,000, although many interventions exceed this figure. Interventions with a cost below $50,000 would indicate a good value, while those above this cut-off indicate less value. Augmenting this excellent test were donor questionnaire inquiries to ferret out behaviors that might predispose a donor to harboring HIV. These initial screens eliminated many infectious persons from the donor pool. However, blood collection centers continued to add tests to improve the sensitivity of their screening for HIV and other viruses, partly to counteract the image that they had been insufficiently diligent in guarding the safety of the blood supply. Their response, while understandable, resulted in increasing costs of infectious marker testing for tiny marginal improvements in blood safety. HIVp24 antigen was added to reduce the window period of infection at a cost of $2.4 million per QALY. Addition of minipool NAT HIV testing with the dropping of HIVp24 antigen testing costs $1.5 million per QALY. The cost when the NAT is run on an individual donor sample is $7.3 million per QALY [8].

In the US, the FDA mandates what tests must be performed on donated blood. It is aided by the Blood Products Advisory Committee (BPAC) composed of physicians, scientists, blood industry representatives, and laymen. This group is charged with evaluating proposed blood safety measures, but told not to consider the associated cost issues. While not obligated to follow the BPAC's recommendations, the FDA usually does. Even before the FDA requires a new safety measure, many blood centers will implement a new testing technology and it will become the de facto standard of care. Other blood centers can choose to adopt the new technology or face the legal liability that comes with not meeting the established standard of care.

Blood centers are in an enviable position in that they can pass the cost of marginal improvements in blood safety on to hospitals. Hospitals do not have the choice of what blood safety measures they would like to implement; that decision has already been made and the price increases have already been set.

Improvements in viral testing, while costly, do provide some benefits. Leukoreduced components have benefits only in selected patient populations [9]. Universal leukoreduction (ULR), however, has been adopted as the single most expensive safety measure to be introduced in the name of blood safety, contributing $30 to $40 cost per red cell unit, without proven benefits for most patients. Theories abound about the *possible* benefits for all such as reduction of immunomodulatory effects of transfusion, but these theories have remained unproved. Transfusion medicine experts are sharply divided on the merits of ULR [10–13]. Nonetheless, blood services in Canada, Great Britain and most blood centers in the US have implemented this costly addition. While the BPAC voted in favor of ULR in 1998, [14] the FDA has not mandated its implementation.

Allocation of resources to insure the pharmaceutical purity of blood may be a laudable goal, but may violate the ethical principle of justice when funds are not available for improvements that would yield greater benefits.

## 4. The Incredible Shrinking Donor Pool

The most recent loss of donors in the US has been in response to FDA donor deferral criteria designed to decrease the risk of transfusion-transmitted variant Creutzfeldt-Jakob Disease (vCJD) [15]. The FDA states that about 5% of donors may be deferred with up to a 10% deferral rate in large coastal cities [16].

This donor deferral was a precautionary measure. In the US, a particularly unfortunate aspect of this deferral is that it impacted persons who had spent more than six months on a military base in Europe between 1980 and 1996, eliminating a substantial number of military and former military personnel. Many of the excluded donors had begun donating during basic training and developed the habit of frequent donation. It is unfortunate that these committed, repeat donors, whom we know tend to have lower rates of transmissible diseases, have been eliminated.

While the likelihood of transmission of vCJD in this group of deferred donors is unknown, we well know that blood centers will have to be more aggressive in recruiting new donors or persuading current donors to donate more often. In the US, the FDA decides what incentives for blood donation are allowed and what incentives are disallowed; they have published draft guidance for industry [17]. The primary criterion for the ability to label a blood product as coming from a volunteer is that no incentive provided can be readily converted to cash. Giving tee shirts or coupons for ice cream is acceptable. Giving tickets to a major college football game is not. Clearly the FDA wants to keep donors from being seduced by incentives that might lead them to lie on the donor questionnaire. One incentive that is deemed acceptable, and that has been used by US blood centers, is lotteries. Donors who donate during a given time period are included in a drawing for a variety of desirable items. Raffled items have included Rolling Stones tickets [18], Caribbean cruises [19], classic sports cars [20], and new automobiles [21]. The FDA states that the value of the item raffled is irrelevant. However, given the large monetary value and desirability of a new automobile or a cruise, some persons donating might be lured to donate solely for their chance at claiming a very valuable prize. So enticed, some persons might be economical with the truth. Abandoning altruism as the primary motivation for donation will likely make the blood supply less safe. It is disingenuous to think that because a practice is not proscribed by a regulatory agency it is therefore safe.

The utilization of incentives raises the issue of nonmaleficence since induced donations may be less safe than totally voluntary donations. However, this principle of “do no harm” competes with the ethical concept of beneficence in that inducements may increase the total blood supply.

## 5. Caving to Political Pressure

As more deferrals are added, there is increasing pressure to examine the validity of older deferrals. One deferral that has generated widespread debate is the permanent deferral of men who have had sex with other men since 1977 (MSM) [22]. There have been allegations of homophobia [23–25], disruption of blood drives [26–28], and exhortations by some to lie on the donor questionnaire regarding sexual activity [29]. This has become a gay-rights issue, and lobbying efforts have been well coordinated. The FDA's BPAC, by a 7 to 6 vote in 2000, voted to maintain the present policy [30]. The scientific studies have not been as ambiguous as the controversy suggests. They have shown that decreasing the ban to five-, one-, or zero-year-deferral would increase the likelihood of HIV being transmitted in the blood supply [31, 32]. While a change would capture some who happen to have been celibate for a certain period, it seems unlikely that potential blood donors, homosexual, or heterosexual, would be celibate for one or five years for the sole purpose of donating blood. One study indicated that only 1 in 3 current MSM-deferred donors would be allowed to donate if the deferral were to be altered to one year and only 1 in 6 would be allowed if the deferral period were to be altered to 5 years, concluding “Our analysis … does suggest that impact on recruitment would be negligible” [33]. 

The AABB has lobbied for replacing the lifetime ban to a one-year deferral [34]. While the American Red Cross (ARC) formerly supported the ban, they have now joined the AABB and America's Blood Centers in asking the FDA to reconsider.

Regarding the lifetime ban, they state:

It does not appear rational to broadly differentiate sexual transmission via male-to-male sexual activity from that via heterosexual activity on scientific grounds neither does it seem reasonable to extend this reasoning to other infectious agents. To many, this differentiation is unfair and discriminatory, resulting in negative attitudes to blood donor eligibility criteria, blood collection facilities, and, in some cases, cancellation of blood drives [35].

Apparently willing to lose more than they have gained, blood centers are advocating changes that will expose blood recipients to higher rates of infectious disease while zealously pursuing expensive infectious disease testing that provides meager marginal improvements. Either they have caved to political pressure or they truly believe what they are proposing is the right thing to do. If the latter is the case, then blood collection centers should advocate an end to another permanent deferral: persons who have ever injected a substance into their arms without a doctor's prescription. Certainly, intravenous drug users who have abstained from using drugs for some period of time should be safe to donate. Or since our testing is so excellent, perhaps active intravenous drug users should be sought out to donate. Our testing has so reduced the window period, it is hardly reasonable to discriminate against these potential donors. The fact that intravenous drug users, active or former, have failed to successfully lobby is not a reason to deny them their “right to donate.” If we are to make our primary concern that we will not hurt anyone's feelings, then we should unabashedly say that donors' rights come first and the recipients will have to pay the price. Creating a benefit for one group—the donors—to the detriment of another—the recipient—violates the ethical principle of justice and nonmaleficence. I should make it clear that I do not support seeking out former intravenous drug addicts and use this example as hyperbole to illustrate that by allowing any high-risk group to donate we are not fulfilling our basic responsibility to recipients. We should not relax criteria on any high-risk group in order to placate deferred donors or activists.

## 6. Ignoring What Could Really Make Blood Transfusion Safer

While our efforts have focused on the purity of the blood in the bag, we have neglected efforts such as ensuring proper patient and blood product identification that could result in much greater gains in terms of overall transfusion safety. Many more patients suffer morbidity and mortality from receiving the wrong blood than those who are infected with transfusion-transmitted viruses [36]. The primary problem is human error, and the solution, like that for reducing viral transmission, is the implementation of new technology. The use of technology such as barcoding, radio frequency identification, and biometrics can help ensure that the correct blood will be transfused to the correct patient [37, 38]. While the cost of implementing new technology is not insignificant, it is primarily one of initial capital investment with rather low marginal costs in the long run. Some improvements such as the use of barrier technology such as the Bloodloc system can actually save money for hospitals [39]. However, with the constantly increasing prices of blood due to implementation of newer, more expensive viral testing procedures, transfusion medicine specialists will likely have a hard time selling the idea of investing more funds into technology for safer transfusion practices to hospital administrators. They will likely perceive the bloated blood bill as representing ample resources being funneled to blood transfusion, and, in the zero-sum game model, the resources for improvements in transfusion practice safety will have been diverted to efforts to increase the sensitivity of the testing of donated blood.

## 7. Enlightening the Public

Much of the driving force for decreasing infectious disease risks of blood transfusion has resulted from public demands. Thanks to the Internet, the general public is becoming more aware of health issues facing them. However, when it comes to blood transfusion, it appears that they do not fully appreciate that other transfusion hazards pose a greater risk than transfusion-transmitted viruses. Getting out the message that there are remediable blood transfusion hazards other than AIDS could help in shifting funding to more cost-effective interventions, but this will not be easy. The literature that transfusionists are well versed in will not be effortlessly translated to the layman. Studies have shown a general lack of health literacy. In one study, the majority of a group of patients, all of whom had at least had the equivalent of a high school diploma, were unable to accurately predict how many times a coin would land heads if flipped 1,000 times [40]. Another study assessing the ability of patients in a city hospital to understand basic instructions such as what it meant to take medication on an empty stomach showed that many did not comprehend the information [41, 42]. If we are to be successful in partnering with the public for safer transfusion, we will have to be creative in how we convey the information to them. Graphic representations of relative risks such as Paling charts may be better received than raw probability estimates. Further research in how to clearly inform those not scientifically versed may elucidate better ways to get our message across.

## 8. We Are Unable to Turn Back the Clock

While one may be free not to make a gift, taking the gift back is not an option. It is possible to not offer a screening test, but it is nearly impossible to withdraw a test that has been previously implemented. An example of this is the serologic test for syphilis (STS) performed on blood products. In 1999, the AABB suggested to the FDA's BPAC that “the requirement for performing an STS on each whole blood donation can safely be eliminated based on thirty years experience” [43]. The AABB pointed out that transfusion-transmitted syphilis had not been recognized in the US in more than 30 years and that the STS had a low efficacy as a surrogate marker for HIV. Nonetheless, the FDA decided to keep the STS.

While studies have shown that some of the newer testing technologies for viral detection may be very cost-ineffective, once implemented, I believe they are here to stay. If a change were proposed, I believe the public and media perception would be that the blood establishment is taking steps to make the blood supply less safe. The take-home message is: be careful what you put in place for you will likely be stuck with it.

Tests have been dropped. The alanine aminotransferase (ALT) test was dropped after a more sensitive test for Hepatitis C (anti-HCV) was implemented [44]. The HIV p24 antigen test was likewise dropped when NAT testing closed the window period of HIV infection more than the antigen testing [45]. So, if a better, more sensitive test is implemented, an older, less sensitive test may be eliminated, but it is unlikely that the climate of the public will allow eliminating a test, however cost-ineffective, without a better one to take its place.

## 9. Lessons for Developing Countries

While developing countries throughout the world may be tempted to mimic the methods employed to improve blood safety utilized by developing countries, they would be wise to be circumspect in emulating all of their practices. In his testimony as Executive Vice President of America's Blood Centers before the HHS Committee on Blood Safety and Availability in April 2001, Dr. Celso Bianco summarizes the problem:

… What do developing countries say they want? The answer is almost unanimous. They want blood systems that are identical to those in the U.S. and in the rest of the developed world … Trainees come to the AABB meeting, look at the technology exhibit, and are mesmerized. They want it all. They return to their countries of origin and dream about setting up blood banks identical to those that they saw abroad. Some actually succeed in doing so. Then they realized that the system depends on the availability of qualified blood donors. This was not part of their training and was not mentioned by the local salesmen pitching technology wares. Trainees did not learn how to recruit volunteer blood donors … The situation is aggravated by the uncritical adoption of U.S. testing standards … I believe that to some degree we bear responsibility for the confusion about blood safety standards around the world. Despite the communication and interaction between regulatory agencies and blood banking organizations among developed countries, we cannot agree among ourselves on procedures for the management of the safety of the blood supply. We are driven by fear, politics and sometimes demagoguery [46].

Developing countries have the opportunity of examining a large body of literature that has been published regarding cost-effectiveness of blood safety measures in developed countries. Critical examination of what has been done in developed countries and what is left to be done could provide developing countries with a blueprint for what measures will bring them the most return for their investment. The prevalence of infectious diseases varies among countries and different choices for different countries are sensible. Blind following of the efforts of developed countries will not likely best serve the interests of those trying to establish basic safety measures. The role of developed countries should be to be candid about which efforts have resulted in the highest benefit and not encourage other countries to believe that developed countries' efforts should be automatically adopted. A recent consensus conference on this issue provides a framework that may be helpful to developing countries [47].

## 10. Summary

Efforts for a zero-risk blood supply have resulted in unintended consequences that have made blood transfusion less safe. Increasing expenditures for better infectious marker testing continue to funnel resources from basic measures such as better patient identification systems. The shrinking donor base has pressured centers to utilize incentives that may entice dishonesty on the donor questionnaire. Our concern for rights of potential donors threatens to increase the risk of HIV for our blood recipients. Bearing in mind the principles of nonmaleficence, beneficence, autonomy, and justice, developed countries need to take a broader view of overall blood transfusion safety and developing countries should learn from their mistakes.
